# HERV-K(HML-2) *rec* and *np9* transcripts not restricted to disease but present in many normal human tissues

**DOI:** 10.1186/s13100-015-0035-7

**Published:** 2015-03-05

**Authors:** Katja Schmitt, Kristina Heyne, Klaus Roemer, Eckart Meese, Jens Mayer

**Affiliations:** Institute of Human Genetics, Center of Human and Molecular Biology, Medical Faculty, University of Saarland, 66424 Homburg/Saar, Germany; José Carreras Research Center, Medical Faculty, University of Saarland, 66424 Homburg/Saar, Germany; Center of Human and Molecular Biology, University of Saarland, 66424 Homburg/Saar, Germany; Sanofi-Aventis Deutschland GmbH, Industriepark Hoechst, K703, Elisabeth Kuhn Street, Frankfurt/Main, 65926 Germany

**Keywords:** Human endogenous retrovirus, Provirus, Transcription, Splicing, HERV-K Rec protein, HERV-K Np9 protein, Retrotransposition, L1 element

## Abstract

**Background:**

Human endogenous retroviruses of the HERV-K(HML-2) group have been associated with the development of tumor diseases. Various HERV-K(HML-2) loci encode retrovirus-like proteins, and expression of such proteins is upregulated in certain tumor types. HERV-K(HML-2)-encoded Rec and Np9 proteins interact with functionally important cellular proteins and may contribute to tumor development. Though, the biological role of HERV-K(HML-2) transcription and encoded proteins in health and disease is less understood. We therefore investigated transcription specifically of HERV-K(HML-2) *rec* and *np9* mRNAs in a panel of normal human tissues.

**Results:**

We obtained evidence for *rec* and *np9* mRNA being present in all examined 16 normal tissue types. A total of 18 different HERV-K(HML-2) loci were identified as generating *rec* or *np9* mRNA, among them loci not present in the human reference genome and several of the loci harboring open reading frames for Rec or Np9 proteins. Our analysis identified additional alternative splicing events of HERV-K(HML-2) transcripts, some of them encoding variant Rec/Np9 proteins. We also identified a second HERV-K(HML-2) locus formed by L1-mediated retrotransposition that is likewise transcribed in various human tissues.

**Conclusions:**

HERV-K(HML-2) *rec* and *np9* transcripts from different HERV-K(HML-2) loci appear to be present in various normal human tissues. It is conceivable that Rec and Np9 proteins and variants of those proteins are part of the proteome of normal human tissues and thus various cell types. Transcription of HERV-K(HML-2) may thus also have functional relevance in normal human cell physiology.

**Electronic supplementary material:**

The online version of this article (doi:10.1186/s13100-015-0035-7) contains supplementary material, which is available to authorized users.

## Background

Human endogenous retroviruses (HERVs) stem from ancient germ line infections by exogenous retroviruses. About 8% of the human genome mass consists of retroviral sequences in *sensu stricto* and sequences with retroviral portions. There are about 40 phylogenetically distinct HERV groups documenting germ line integration, that is, provirus formations by different ancient exogenous retroviruses millions of years ago. Re-infections and intracellular amplifications often increased numbers of proviruses per HERV group for limited evolutionary time periods following initial integration events. Most HERV groups no longer encode former retroviral proteins due to long time presence in the genome and thus accumulation of nonsense mutations including smaller and larger indels. Some retroviral proteins, in particular Envelope (Env), have been conserved during evolution to contribute important Env-mediated functions such as fusion of cell membranes [1-4].

**Figure 1:**
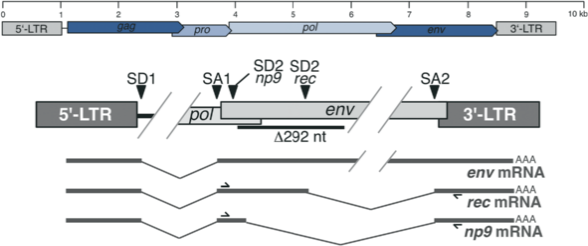
**Schematic of HERV-K(HML-2) provirus and splicing of** ***rec*** **and** ***np9*** **mRNAs.** Irrelevant proviral regions are omitted. *rec* mRNA is generated by a second splicing event of *env* mRNA removing most of the *env* gene region. Both *rec* and *np9* mRNA utilize the same splice acceptor site just upstream of the 3′LTR. Because SD2 for *rec* mRNA is located within the 292-bp sequence missing in HML-2 type 1 proviruses, an alternative *np9*-specific SD2 is used instead also resulting in a translation frameshift when spliced to exon 3. Location of PCR primers used for amplification of *rec* and *np9* mRNA/cDNA is indicated. Note that the lower provirus map is not drawn to scale. LTR, long terminal repeat.

The so-called HERV-K(HML-2) group (in short, HML-2) includes a number of evolutionarily young proviruses, some of which formed in the human lineage after the evolutionary split of human from chimpanzee about 6 million years ago. Especially the young HML-2 loci often harbor open reading frames (ORFs) for retroviral proteins such as Gag, Protease, Polymerase, and Envelope. Analyses of HML-2 proviral transcripts had identified typical retroviral splicing events generating an *env* mRNA and a sub-spliced *env* mRNA, originally named *cORF* and later re-named *rec*, with most of the envelope coding sequence removed. Historically, HML-2 proviruses have been divided into type 2 loci, the transcripts of which can be sub-spliced to *rec* mRNA, and type 1 loci that lack a characteristic 292-bp sequence located about 50 bp into the *env* coding sequence [5,6]. Lack of the 292-bp sequence in type 1 loci impairs sub-splicing of *env* mRNA to *rec* mRNA because of lack of the *rec* splice donor (SD) site located within the deleted region. Instead, a SD site just upstream of the 292-bp deletion is now employed in combination with a splice acceptor (SA) site located at the 3′ end of *env* that is the same SA for splicing of transcripts from type 1 and type 2 loci. Such spliced transcripts derived from HML-2 type 1 loci have been named *np9* [7,8] (Figure 1).

Clinical relevance of HML-2 transcription and proteins has been investigated in the context of various human diseases. Especially germ cell tumors (GCT) display strongly upregulated HML-2 transcription and expression of HML-2 proteins already in early stages of tumor development. GCT patients display strong antibody titers against HML-2 Gag and Env proteins at the time of tumor detection (reviewed in ref. [2]).

Both *rec* and *np9* mRNA can encode proteins with potentially important cellular functions that may be relevant to disease development. HML-2 Rec protein is basically a functional homologue of HIV_Rev_ protein [9-13]. Nude mice transgenic for Rec protein develop lesions reminiscent of testicular carcinoma *in situ* [14]. Rec protein was shown to interact with several functionally relevant cellular proteins such as promyelocytic zinc finger protein (PLZF), testicular zinc finger protein (TZFP), Staufen-1, and human small glutamine-rich tetratricopeptide repeat protein (hSGT). Np9 protein was shown to interact with PLZF and ligand of Numb protein X (LNX). All of those interactions may have important cellular consequences depending on cellular context [15-20].

Several recent studies have identified a number of HML-2 loci transcribed in various disease as well as normal conditions by assigning specifically generated HML-2 cDNA sequences to genomic HML-2 loci employing characteristic sequence differences between HML-2 loci, with transcription patterns varying considerably between conditions (for instance, see [21-26]). Several of the transcribed HML-2 loci can, in principle, encode *rec* or *np9* mRNA. We have previously analyzed HML-2 loci specifically for coding capacity for *rec* mRNA and protein by analysis of HML-2 locus sequences for features required for *rec* mRNA splicing and presence of a Rec ORF within predicted mRNA sequences. We also had identified a number of HML-2 loci generating *rec* mRNA by means of cDNA sequence assignments to genomic HML-2 loci [21,27].

The role(s) of HML-2 Rec and Np9 proteins in human biology is still little understood. As various HML-2 loci are also transcribed in normal human tissue types, it is conceivable that HML-2 proteins also exert biological functions apart from potential roles in disease development. To contribute to a better understanding of a potential biological relevance of HML-2 Rec and Np9, we investigated presence of *rec* and *np9* mRNA in a collection of normal human tissue types and identified HML-2 loci generating *rec* and *np9* mRNA. We also identified additional HML-2 loci not present in the human reference genome sequence and additional splicing variants of HML-2 transcripts potentially encoding HML-2 protein variants in the course of our studies.

## Results

### Identification of HERV-K(HML-2) *rec* and *np9* mRNA in normal human tissues

Recent findings indicated transcription of HERV-K(HML-2) loci in various human cell and tissue types. Several proteins encoded by some HML-2 loci are considered to be involved in the development of some diseases, among them HML-2 Rec and Np9 proteins in the development of certain tumor types. Carrying on the identification of transcribed HERV and especially HML-2 loci in various disease and normal conditions, we were now interested in whether there are *rec* and *np9* mRNAs in normal human tissues and, if so, from which HML-2 loci those *rec* and *np9* mRNAs were generated.

To identify *rec* and *np9* mRNA, we made use of a multiple tissue cDNA panel that included cDNAs from 16 different tissue types (15 actual tissues and peripheral blood leukocytes (PBL), henceforth all designated as ‘tissues’ for the sake of simplicity). We amplified *rec* and *np9* mRNA-derived cDNA by using PCR primers located within exons 2 and 3 of HML-2 proviral full-length transcripts (Figure 1) and considering sequence variations between HML-2 loci within PCR primer binding regions to compensate for potentially suboptimal amplification of respective cDNAs.

**Figure 2:**
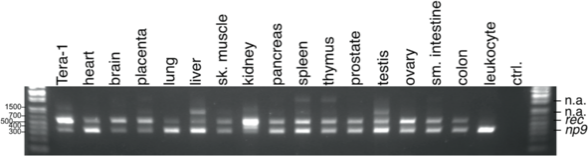
**Gel electrophoretic separation of** ***rec-*** **and** ***np9*** **-specific PCR products amplified from a panel of cDNAs.** Generated from 15 normal human tissues and peripheral blood leukocytes. Expected product sizes were *ca.* 580 and 360 bp for *rec* and *np9*, respectively. cDNA generated from GCT-derived Tera-1 cell line, known to strongly express HML-2 both on the RNA and protein level (for instance, see ref. [28]), served as a positive control. ‘ctrl.’ indicates a PCR control reaction without template DNA. A faint band representing *rec*-specific PCR product from PBL is not properly reproduced. ‘n.a.’ indicates additional PCR products of approx. 1 kb in the liver and testis and approx. 2.2 kb in the spleen and the thymus not further investigated in this study.

PCR products of *ca.* 580 bp indicative of *rec* mRNA could be amplified from all 16 tissue cDNAs, with amplification from cDNA from PBL resulting in only a faint band after gel electrophoresis. PCR products of *ca.* 360 bp indicative of *np9* mRNA could be amplified from all 16 tissue cDNAs as well, with amplification from cDNA from PBL producing a relatively strong PCR product (Figure 2). PCR products of *ca.* 1 kb amplified from liver and testis cDNAs and *ca.* 2.2 kb amplified from spleen and thymus cDNAs were not further regarded in this study. Taken together, *rec* and *np9* mRNA appeared to be present in all tissue types examined in this study.

### Identification of *rec* and *np9* mRNA encoding HERV-K(HML-2) loci

We then identified HML-2 loci having generated those *rec* and *np9* mRNAs. To do so, we cloned *rec* and *np9* mRNA representing PCR products and sequenced inserts from randomly selected plasmid clones. We then assigned resulting cDNA sequences, on average 41 (min. 29, max. 46) per tissue type, to specific HML-2 type 1 and type 2 loci in the human reference genome sequence by means of characteristic sequence differences between the various HML-2 loci. Despite the rather short-sized PCR products, thus short cDNA sequences (excluding primer regions), there was a sufficient number of sequence differences between relevant exon regions of HML-2 loci for unambiguously assigning generated *rec* and *np9* cDNA sequences to loci. For *rec* mRNA-derived cDNAs, only two HML-2 loci in chromosome 1 were identical in sequence for the regarded exon regions, and respective *rec* transcripts could thus, in principle, not be assigned to either one of them (Additional file 1: Figure S1).

We identified *rec* transcripts originating from, in total, nine different genomic HML-2 loci. Some tissue types (lung and colon) appeared to contain *rec* mRNA from up to five different HML-2 loci, while in kidney tissue, *rec* transcripts originated from only one HML-2 locus. Other tissue types displayed intermediate numbers of transcribed *rec* mRNA coding loci. *rec* transcripts from two HML-2 loci in chromosome 2q32.1 and 5q15 were found in 15 and 13, respectively, of the examined tissues (Table 1). As we will describe below, several of those loci are special with regard to *rec* mRNA.

**Table 1:** **Summary of absolute numbers of** ***np9*** **and** ***rec*** **representing cDNA sequences assignable to HERV-K(HML-2) loci** ^**a**^

**Type 1/2 loci**	**HGNC**	**Band**	**Heart**	**Heart tot. RNA**	**Brain**	**Brain tot. RNA**	**Placenta**	**Lung**	**Liver**	**Skeletal muscle**	**Kidney**	**Pancreas**	**Spleen**	**Thymus**	**Prostate**	**Testis**	**Ovary**	**Small intestine**	**Colon**	**Colon tot. RNA**	**Leukocyte**	**ORF**	**mRNA**
chr1:75615359-75621731	*ERVK-1*	1p31.1					1					1										Y	*np9*
chr1:153863081-153872260	*ERVK-7*	1q22	1	*7*			11	15	21	2	1	1	22	11	4	7		10	8	*6*	24	Y	
chr1:158927199-158936430	*ERVK-18*	1q23.3												3			1		1		2		
chr3:102893427-102902549	*ERVK-5*	3q12.3	1	*10*	6	*1*		7	3	1	2	1	1	2	4	7		3	5	*12*		Y	
chr3:114225814-114234972	*ERVK-3*	3q13.2													1							Y	
chr4:166136289-166143518	-	4q32.3														1							
chr22:17306187-17315361	*ERVK-24*	22q11.21			1																	Y	
HERV-K111			16	*6*	10	*5*	2		2	5	6	1		2	2		1	3	4	*11*			
Venter locus			1	*13*	1		6	2		3	4	15			5		13	5	3				
Not assignable *np9*-like			15	*25*	7	*3*	4		2	12	4	5	2	4	12	3	8	5	4	*18*			*rec*
chr2:187093879-187095344	*ERVK-30*	2q32.1	10	*13*	2	*2*	6	5	4	4		13	13	12	3	1	5	4	8	*28*	17		
chr3:127091992-127101129	*ERVK-4*	3q21.2				*1*												1		*1*		Y	
chr5:30522516-30531962	-	5p13.3													1							Y	
chr5:92818136-92819668	*ERVK-31*	5q15	2	*8*	13	*89*	10	11	1			4	7	1	11	7	10	9	7	*12*			
chr6:78483381-78492802	*ERVK-9*	6q14.1						1		15												Y	
chr7:4588583-4606557	*ERVK-6*	7p22.1						1							1				2			Y	
chr10:101570559-101577735	*ERVK-17*	10q24.2					1		8			1				3			1			Y	
chr12:57007509-57016965	*ERVK-21*	12q14.1		*3*			2	1			20			1			1	1	1			Y	
chr19:32820338-32829201	*ERVK-27*	19q12			2																	Y	
Total # of sequences			46	*85*	42	*101*	43	43	41	42	37	42	45	36	44	29	39	41	44	*88*	43		

^a^Normal tissue types from which cDNA sequences were generated are indicated on the top. Values in italics indicate results from experiments starting from total RNA instead of pre-made cDNA specifically for heart, brain, and colon tissue (see paper text). HML-2 loci including chromosomal coordinates in the hg18 human reference genome sequence are indicated in the left-most column, followed by HUGO Gene Nomenclature Committee (HGNC)-assigned locus designations [29] and location in chromosomal bands. Total numbers of analyzed cDNA sequences per tissue are indicated at the bottom. *np9*-like cDNA sequences unambiguously assignable to recently described HERV-K111 and Venter locus sequences not present in the human reference genome sequence (see text for details) are given separately. *np9*-like cDNA sequences not assignable to any of the listed or other HML-2 type 1 locus sequences are also given separately. Presence of ORFs for Np9 or Rec protein (‘Y’) in predicted mRNA sequences derived from the various HML-2 loci is indicated. See Figure 5 for actual protein sequences.

We identified *np9* transcripts originating from, in total, seven different HML-2 loci present in the human reference genome sequence. As for *rec* mRNA, *np9* mRNA originated from variable numbers of HML-2 loci depending on tissue type. *np9* mRNA transcripts from HML-2 loci in chromosomes 1q22 and 3q12.3 were found in 14 and 13, respectively, of the examined tissues (Table 1). Two additional *np9* mRNA encoding HML-2 loci, from which transcripts were identified in many tissues and which are not present in the human reference genome sequence, are described below.

Taken together, our results indicate that *rec* or *np9* mRNA originated from at least 18 different HML-2 loci in various normal human tissue types.

### An additional HML-2 locus formed by L1-mediated retrotransposition of *rec* mRNA

**Figure 3:**
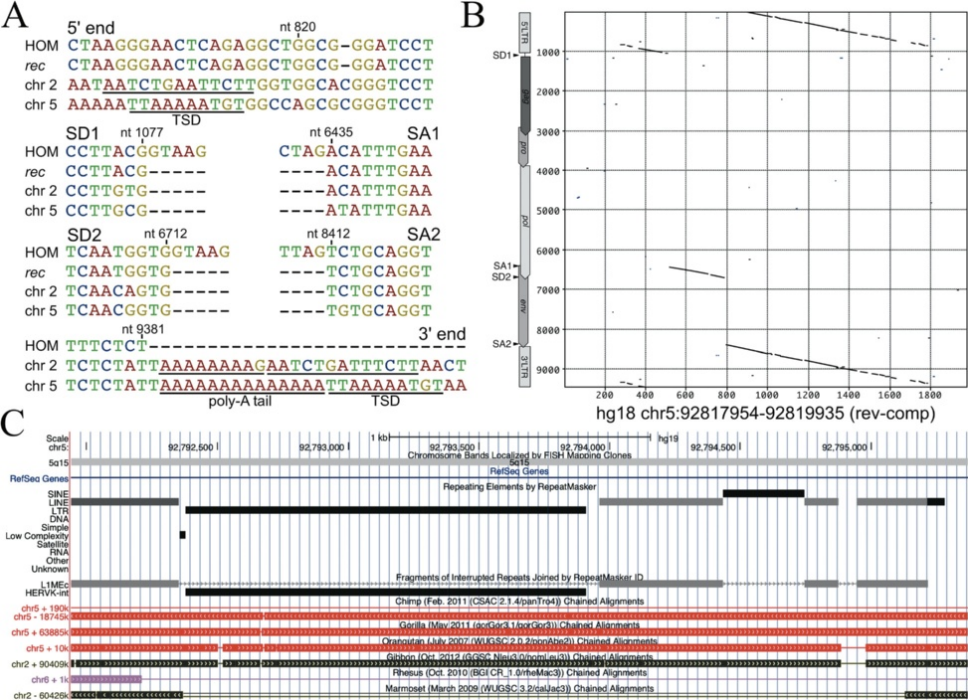
**A HML-2 locus in human chromosome 5q15 once formed by L1-mediated retrotransposition of** ***rec*** **mRNA. (A)** Multiple sequence alignments of relevant regions of the HERV-K(HML-2.HOM) proviral sequence (GenBank acc. no. AF074086; ref. [31]), a *rec* mRNA sequence (GenBank acc. no. X72790; ref. [8]), a recently described retrotransposed *rec* mRNA in human chromosome 2q32.1 [21], and the HML-2 locus in human chromosome 5q15 are shown, depicting target site duplications, *rec*-typical intron/exon boundaries and poly-A tails immediately upstream of the 3′TSD of the chromosome 2q32.1 and 5q15 loci. See ref. [21] for a more detailed description of the chromosome 2q32.1 locus. **(B)** A dot plot matrix comparison of the chromosome 5q15 locus sequence (reverse complemented) with the HERV-K(HML-2.HOM) reference sequence is shown with proviral regions indicated for the latter, likewise depicting the *rec*-typical proviral regions present for that HML-2 locus. **(C)** Presence of the HML-2 locus in the homologous regions of human chromosome 5q15 in the genomes of chimpanzee, gorilla, orangutan, gibbon, and lack in rhesus monkey (the homologous HML-2 locus also lacking in baboon is not shown). Marmosets, as new world monkeys, generally lack HERV-K(HML-2) homologous sequences. LTR, long terminal repeat; SA, splice acceptor; SD, splice donor; TSD, target site duplication.

We have previously reported a HML-2 locus located in chromosome 2q32.1 that was formed by L1-mediated retrotransposition of a *rec* mRNA [21]. In the present study, that locus was found to be transcribed in almost all examined normal human tissues (Table 1). We now identified an additional HML-2 locus located in chromosome 5q15 (hg18; chr5: 92818136–92819668), also transcribed in almost all of the investigated tissues (Table 1), that very likely was also formed by L1-mediated retrotransposition of a *rec* mRNA. The 1.5-kb-long locus is flanked by target site duplications (5′-TTAAAAATGT-3′) typical of an L1 target site consensus sequence [30] with a poly-A tail and a poly-A signal located (more) upstream of the locus’ 3′ end. Apart from an approximately 250-bp 5′ truncation of the retrotransposed mRNA, portions typically missing from a full-length proviral HML-2 sequence are those likewise not present in a *rec* mRNA and boundaries of missing sequence portions coincide with known splice donor and acceptor sites of *rec* mRNA. Also, those sites are basically identical with the ones of the retrotransposed locus in chromosome 2q32.1. The locus in chromosome 5q15 is evolutionarily old as it is also present in the homologous genome regions of chimpanzee, gorilla, orangutan, and gibbon, but missing in rhesus, baboon (data not shown), and the common marmoset (Figure 3). The latter, as a new world primate, is lacking HML-2 homologous sequences entirely. The locus in chromosome 5q15 does not encode a Rec(−like) protein due to a stop codon 20 triplets into the coding sequence.

### True transcription of two retrotransposed HML-2 loci

We employed for amplification of *rec* and *np9* transcripts a pre-made panel of cDNAs. As opposed to true splicing events, the two retrotransposed HML-2 loci in chromosomes 2q32.1 and 5q15 would produce identically sized PCR products when amplified from mRNA/cDNA or genomic DNA. We were therefore concerned that amplified PCR products assignable to those two loci were due to traces of genomic DNA present in the pre-made cDNAs, thus conceivably a false indication of those two HML-2 loci being transcribed in the examined tissues. To investigate this further, we generated cDNA from commercially available total RNA from three normal human tissues, specifically heart, brain, and colon, following own previously established protocols for rigorous DNA removal and including strict controls for DNA contamination [21]. We assigned, on average, 91 cDNA sequences from each of the three tissue RNAs to genomic HML-2 loci. Overall, when taking higher numbers of cDNA sequences generated per tissue into account, we obtained very similar numbers regarding transcribed HML-2 loci when compared to the pre-made cDNA panel, especially regarding cDNA sequences assignable to the loci in chromosomes 2q32.1 and 5q15 (Table 1). It thus appears that the two retrotransposed HML-2 loci are truly transcribed in quite a number of normal human tissue types.

In accord with an HUGO Gene Nomenclature Committee initiative [29], the two HML-2 loci in chromosomes 2q32.1 and 5q15 were designated *ERVK-30* and *ERVK-31*, respectively.

### Transcription of HML-2 loci not present in the human reference genome sequence

Generated cDNA sequences regularly included *np9* mRNA-like sequences that could not be assigned to HML-2 type 1 loci in the human reference genome sequence. At first glance, one population of sequences was most similar to the *ERVK-5* locus in chromosome 3q12.3, yet almost all those sequences uniformly displayed eight different nucleotide positions to that locus. Further analysis provided evidence that about half of those cDNA sequences were very likely transcribed from recently reported HERV-K(HML-2) loci not present in the human reference genome sequence. Specifically, out of 280 cDNA sequences deemed unassignable to HML-2 loci in the human reference genome, a subset of 76 cDNA sequences were identical to the recently reported HERV-K111 sequence (GenBank acc. no. GU476554; ref. [32]) along the comparable proviral regions and thus were presumably transcribed from the HERV-K111 locus. Another subset of 71 cDNA sequences were identical to a recently reported, 4214-bp-long sequence entry consisting of a partial HERV-K(HML-2) type 1 locus (GenBank acc. no. ABBA01159463; ref. [33]) (DNA donor: J. Craig Venter; henceforth named ‘Venter locus’) and were thus most likely transcribed from the Venter locus (Additional file 1: Figure S2). Another subset of 133 unassignable sequences was neither identical to HERV-K111 nor the Venter locus.

**Figure 4:**
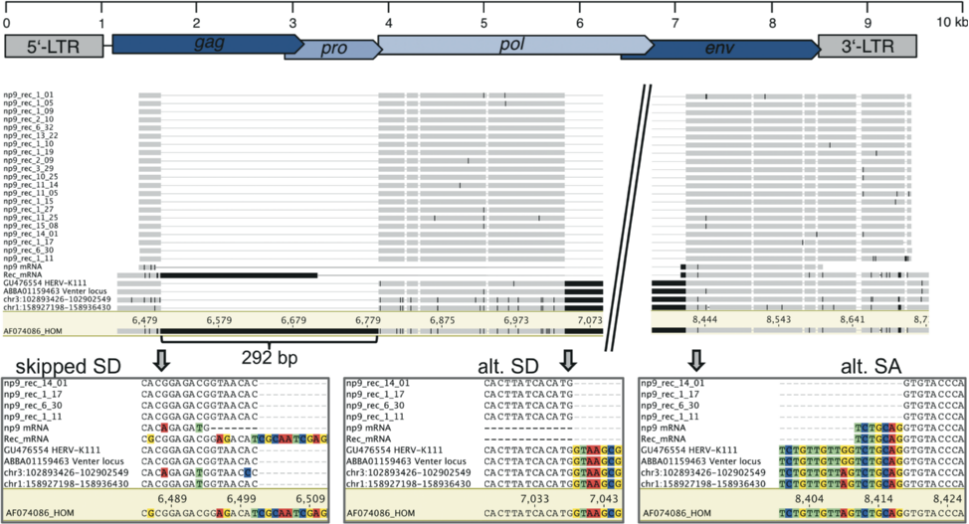
**Excerpts from a multiple sequence alignment of an alternative exon from one or several HERV-K(HML-2) type 1 loci.** The utilized SD site for the *np9* intron 2 (‘alt. SD’) is located more downstream compared to the canonical np9 SD2 (‘skipped SD’). The 3′ end of intron 2 is also located 7 nt more downstream compared to the canonical *np9*/*rec* mRNA SA2 (‘alt. SA’). Several sequences have been included for comparison: canonically spliced ‘np9_mRNA’ and ‘Rec_mRNA’ [7,8], corresponding sequence portions from the HERV-K111 provirus [32], the Venter locus (GenBank acc. no. ABBA01159463; ref. [33]), and the HERV-K(HML-2.HOM) (type 2) provirus (GenBank acc. no. AF074086; ref. [31]) (nt positions of that sequence are given as reference), HML-2 loci *ERVK-5* and *ERVK-18* in human chromosomes 3 and 1 (nt positions according to hg18) as most closely related, yet, clearly different HML-2 proviruses (see the paper text). Most of the *np9*/*rec* intron 2 has been omitted from the figure, as indicated. Nucleotide positions deviating from the consensus sequence of included sequences are highlighted in black. Enlarged alignment regions shown at the bottom depict usage of a different SD2 site compared to *np9* mRNA (left and center) and usage of a different SA2 compared to *rec* and *np9* mRNA. Note the mutated SA2 site (AG → GG) in the HERV-K111 and Venter locus sequences (see the paper text). A HERV-K(HML-2) provirus map with locations of ORFs is shown at the top. LTR, long terminal repeat; SA, splice acceptor; SD, splice donor.

A fourth cDNA sequence population was most similar to the *ERVK-18* locus in human chromosome 1q23.3, yet uniformly displayed 25 differing nucleotides to that locus. That sequence population harbored an additional 245 nt compared to the amplified *np9* cDNA-derived PCR product. The difference in length was due to a SD signal located 252 nt downstream within the *env* gene region and a different SA2 located 7 nt downstream from the canonical *rec*/*np9* SA2 (see below). Also, those sequences lacked the 292-bp sequence discriminating HML-2 type 1 and type 2 loci, so that they cannot be interpreted as *rec*-like mRNA transcribed from a HML-2 type 2 locus (Figure 4). The sequences displayed between 2- and 5-nt differences along the comparable 567 nt of cDNA sequence to the Venter locus and to HERV-K111. It is conceivable that those 245-bp longer cDNA sequences, compared to *np9* mRNA, represent alternatively spliced transcripts from HML-2 type 1 loci, potentially from sequence alleles of HERV-K111, several of which have been reported recently [34], or an allele of the Venter locus, or other hitherto unknown sequence or presence/absence alleles of HML-2 loci, several of which were partially described recently [35] (see also the Discussion section).

All of the abovementioned sequences appeared to have been spliced differently from *np9* mRNA. Specifically, the SA site of intron 2 (removing most of the envelope coding region) was located 7 bp downstream from the canonical *rec* and *np9* mRNA SA2 site [7,8]. This was most likely due to both HERV-K111 and the Venter locus harboring a mutated SA2 (5′-TGTT*AG*TCTG-3′ → 5′-TGTT*GG*TCTG-3′) and a SA signal located 7 bp downstream (5′-CTGC*AG*GTGT-3′) being used instead (Figure 4).

Taken together, based on detected sequence similarities, our analysis provided evidence for HERV-K111 and the Venter locus being transcribed and encoding *np9*-like mRNA in various normal human tissue types. It was unclear whether one or several alleles of those two loci or another HML-2 type 1 locus encodes an alternatively spliced, 245-bp longer mRNA.

### Coding capacity of *rec* and *np9* mRNAs

**Figure 5:**
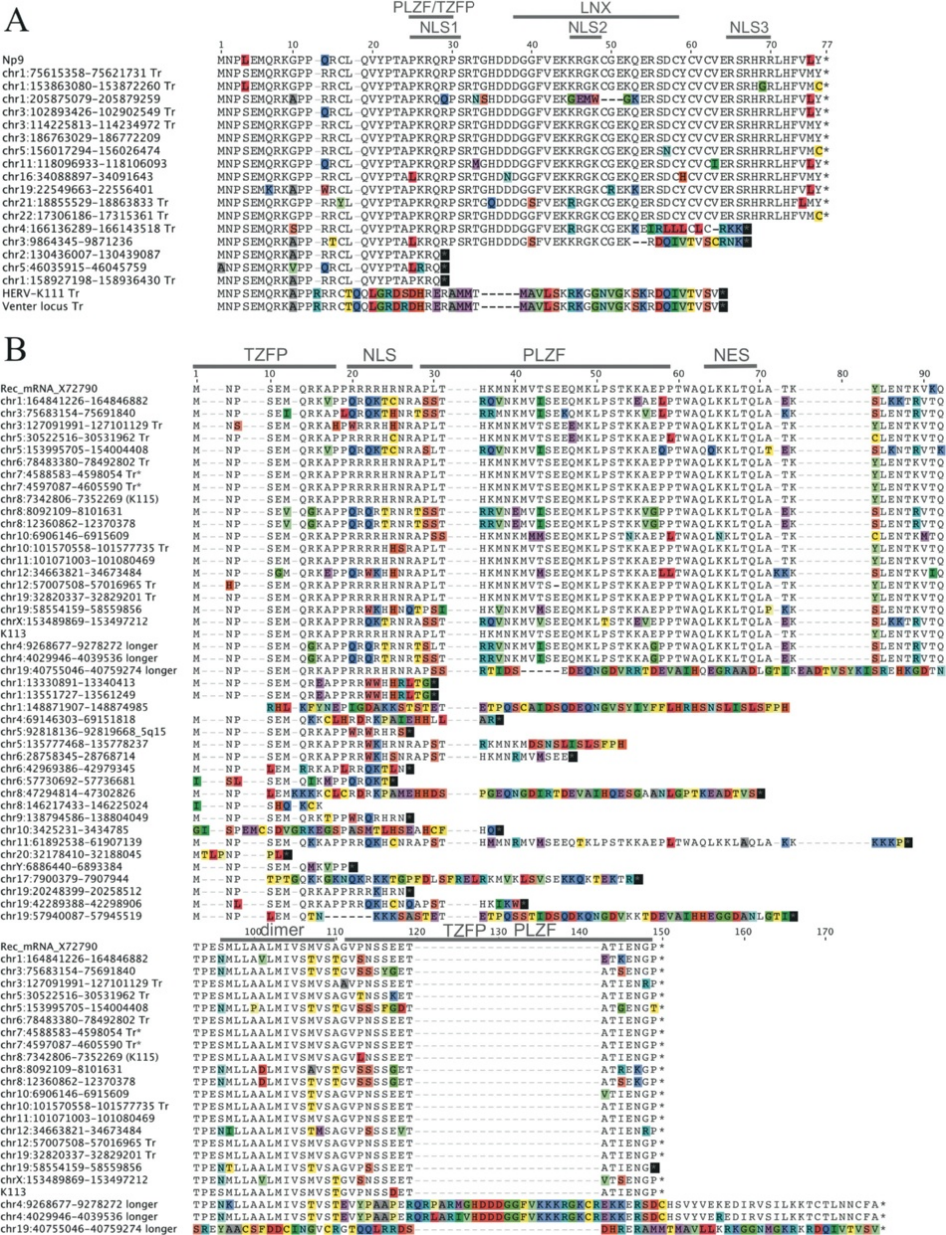
**Multiple sequence alignments of conceptual translations of HML-2 type 1 and type 2 Np9 and Rec ORFs.** HML-2 loci potentially encoding Np9 **(A)** or Rec **(B)** protein and loci encoding only shorter proteins are shown. Chromosomal positions are given for each locus with respect to the human hg18 reference genome sequence. ‘Tr’ indicates that the locus was identified as transcribed in the present study. Protein sequences of a previously identified Np9-encoding mRNA [7] and a Rec-encoding mRNA (GenBank acc. no. X72790; ref. [8]) are given on the top of each alignment. Previously reported Np9 and Rec functional domains are indicated in each alignment. Full-length protein-encoding HML-2 loci are given in chromosomal order followed by shorter Np9-like and Rec-like ORFs. Proteins encoded by type 1 loci HERV-K111 and the Venter locus (see text) are included in the Np9 alignment. Amino acid positions different among each other are highlighted. NES, nuclear export signal; NLS, nuclear localization sequence; PLZF, promyelocytic zinc finger protein; TZFP, testicular zinc finger protein.

We analyzed whether transcribed *rec* and *np9*(−like) mRNAs also have potential to encode Rec and Np9 proteins. We have previously analyzed the capacity of genomic HML-2 type 2 loci to encode Rec protein and have identified a number of HML-2 loci encoding *rec* mRNA [27]. We now analyzed in a similar fashion the capacity of HML-2 type 1 loci to potentially encode Np9 protein by (i) presence of canonical SD and SA sites, (ii) and an ORF for Np9 protein within the predicted mRNA sequence. We identified a total of 12 HML-2 type 1 loci to potentially produce a spliced mRNA and to harbor an ORF for Np9 protein as previously reported in size [7]. Several of the resulting Np9 proteins displayed amino acid differences compared among each other (Figure 5). For instance, a Np9 protein potentially encoded by a locus on chromosome 1 (hg18: 205875079–205879259) would harbor a deletion of three amino acids and additional amino acid differences overlapping with previously reported nuclear localization and LNX protein interaction domains [27]. Other HML-2 type 1 loci could potentially only encode Np9-like proteins about ten or more amino acids shorter than full-length Np9, or being similar to Np9 only within the N-terminal third of a (shorter) protein. This is also the case for proteins potentially encoded by HERV-K111 and the Venter locus that are identical with the canonical Np9 protein sequence only for the N-terminal 15 aa (Figure 5). Notably, several of the potentially protein encoding HML-2 type 1 loci were found transcribed in various normal human tissues.

Similar to our study from a decade ago [27], we re-analyzed for the present study HML-2 type 2 loci potentially encoding Rec protein by examining sequence features required for the splicing of *rec* mRNA and the translation of a Rec protein. Our re-analyses identified ORFs for Rec protein presumably in up to 19 HML-2 type 2 loci, among them the two polymorphic HERV-K113 and HERV-K115 loci [36]. Two other HML-2 loci on human chromosome 4 (hg18: 4029946–4039536 and 9268677–9278272) may potentially encode much longer, Rec-like proteins if they were transcribed. Several other loci only harbor (very) short Rec protein ORFs. The potential full-length Rec proteins display various amino acid differences when compared among each other (Figure 5). As for Np9, several of the potentially Rec protein encoding loci were found transcribed in various normal human tissues.

## Discussion

Several lines of evidence suggest biological significance of HERV-K(HML-2)-encoded proteins Rec and Np9 in the development of tumor diseases, for instance, germ cell tumors and melanoma (for reviews, see [2,37]). However, biological roles of those proteins still need to be investigated in much more detail. Since transcripts from several HML-2 loci have been identified in non-tumor and even normal tissues, it is also conceivable that Rec and Np9 exert biological roles in normal human tissues. We therefore investigated in this study whether there are *rec* and *np9* mRNA transcripts also in normal human tissues. Our strategy for identification of such transcripts involved the amplification of a PCR product encompassing the second and third exons of *rec* and *np9* mRNA. Sequence differences within primer binding regions were also considered to potentially demonstrate transcripts from sequence-diverged HML-2 loci (see also ref. [21]). Contrary to previous studies (for instance, see [21,38]), this study does not allow for estimating relative transcript levels for different HML-2 loci as *rec* and *np9* mRNA representing PCR products were isolated and cloned in a combined fashion thus potentially falsifying relative frequencies of *rec* and *np9* mRNA-derived sequences and transcribed loci. It is also conceivable that full-length transcripts from some HML-2 loci are spliced more efficiently to *rec* or *np9* mRNA than those from other loci. Nevertheless, higher numbers of cDNA sequence derived from particular loci may hint towards higher transcript levels or more efficient splicing of full-length transcripts from those loci. Also, amplified cDNA sequences do not fully document the structure of the actual *rec* and *np9* mRNA sequences as we amplified exons 2 and 3 encompassing intron 2, disregarding exon 1 and intron 1 of *rec* and *np9* mRNAs. It is therefore, in principle, possible that for some HML-2 loci transcript, regions outside of the examined regions are spliced in some non-canonical way, though Rec and Np9 protein coding regions appear to be spliced properly (see also below).

Independent of that, *rec* and *np9* mRNA appear to be present in quite a number of human tissue types as indicated by respective PCR products amplified from 16 normal human tissues investigated in this study.

Our assignments of *rec* and *np9* cDNA sequences to HML-2 loci also indicate that at least 18 different HML-2 loci can be transcribed in normal human tissues and very likely encode *rec* or *np9* mRNAs. Additional transcribed loci encoding *rec* or *np9* may be identified, and tissue-specific patterns of such *rec* or *np9* mRNA encoding loci may be identified when much higher numbers of cDNA sequences than in this study are assigned to HML-2 loci.

Our assignment of cDNA sequences to HML-2 loci also lends support to peripheral blood leukocytes not being the sole source of *rec* and *np9* mRNAs in the various tissues as all tissues would then have displayed a PBL-typical basic pattern of transcribed HML-2 loci. However, HML-2 loci identified as transcribed and encoding *rec* and *np9* mRNAs in PBL are quite different from locus patterns observed for the various tissues. As tissues are always composed of various cell types, it remains to be investigated which cell types in the regarded tissues actually produce *rec* and *np9* mRNAs. Different scenarios are conceivable. For instance, some HML-2 loci may be transcribed and produce a subset of *rec* and/or *np9* mRNAs in some cell types within a particular tissue, while other cell types within that tissue contribute a different mRNA subset because of other HML-2 loci being transcribed in those cells. The proportions of HML-2 loci transcribed in different cell types could differ considerably between tissue types. Eventually, cell type-specific transcription patterns will have to be established.

As for Rec and Np9 protein levels, it is currently not known how efficiently *rec* and *np9* mRNAs are translated into respective proteins, how stable those proteins are in normal human cells, and whether there is protein translated in one or the other tissue/cell type at all. Cell culture-based experiments demonstrated a relatively stable HML-2 Rec protein with a half-life >8 h [39]. Np9 protein seems much shorter-lived in cell culture experiments [18,19]. Nevertheless, little appears to be known about the expression of Rec and Np9 proteins in normal and diseased conditions. Rec protein expression was reported in some melanoma tissue samples, but not in melanocytes or normal lymph nodes [40,41] and in normal synovial, rheumatoid arthritis, and osteoarthritis specimens [42]. Np9 protein was identified in EBV-positive Raji cells that are derived from a Burkitt’s lymphoma, and in an EBV-transformed human lymphoblastoid cell line, IB4 [43]. The detection of Rec and Np9 proteins was accompanied in those studies by detection of *rec* and *np9* mRNAs. Therefore, *rec* and *np9* mRNAs present in normal human tissues imply presence of Rec and Np9 protein in normal human tissues. However, specially designed studies on Rec and Np9 protein levels, half-life, cellular distribution, and so on, in normal human cells will be required. Well-suited Rec- and Np9-specific antibodies appear crucial for such protein studies including Western blot and immunohistochemistry and immunocytochemistry for examination of tissue and cellular distributions, respectively. It seems unclear whether current Rec and Np9 antibodies will be fully suited for such studies especially when considering that several HML-2 protein variants with amino acid sequences and protein sizes very similar to Rec and Np9 proteins have been described recently (for instance, see [21,39]) and in this study.

Our analysis of transcribed HML-2 loci furthermore identified two loci (designated *ERVK-30* and *ERVK-31*), both once formed by retrotransposition of *rec* mRNA by L1 machinery, as transcribed in normal human tissues. Locus *ERVK-30*, located in chromosome 2q32.1, has already been described before [21]. The present study identified another such locus, *ERVK-31*, located in chromosome 5q15, that is due to L1-mediated retrotransposition as it displays typical hallmarks of that process and is identical regarding exon-intron junctions compared to the *ERVK-30* locus in 2q32.1 and *rec* mRNA. The *ERVK-31* locus in chromosome 5q15, located central within a ~57-kb intron of the *NR2F1 antisense RNA 1* (*NR2F1-AS1*) gene producing a non-coding RNA, is about as evolutionarily old as the *ERVK-30* locus in 2q32.1; none of the two loci is present in the homologous regions of the rhesus monkey and baboon genomes but both homologous loci are present in the genomes of subsequent primate species.

Evidence for both loci being transcribed was not due to artifactual amplification from contaminating DNA potentially still present in employed cDNA tissue panels, as demonstrated by our control experiments from total RNA from three selected normal tissues. Sequence data from, for instance, the ENCODE project provide additional support for the retrotransposed *rec* mRNA loci *ERVK-30*/2q32.1 and *ERVK-31*/5q15 being transcribed. Numerous single-pass and paired-read RNA-seq reads generated from various cell lines and normal cell types were mapped to the two loci’s sequence portions (data not shown; ref. [44]). We thus describe here the second instance of a HML-2 *rec* mRNA that was retrotransposed by L1 machinery and is now transcribed by a hitherto unknown promoter active in many human tissues. Contrary to the retrotransposed *rec* mRNA locus in chromosome 2q32.1, the locus in chromosome 5q15 does not appear to encode a Rec-like protein as it harbors a stop mutation about 20 triplets and a frameshift about 63 triplets into the Rec coding sequence.

Our study presumably identified in various human tissues transcripts from two recently described HML-2 loci that are not present in the human reference genome sequence. One population of *np9* cDNA sequences was identical to the recently described HERV-K111 provirus [32]; another population of *np9* cDNA sequences was identical to sequence portions in GenBank acc. no. ABBA01159463, a sequence identified in the genome of J. Craig Venter, that consists of HML-2 *pol*, *env*, and 3′ long terminal repeat (LTR) sequence portions, starting at *ca.* nt 5000 relative to the HERV-K(HML-2.HOM) proviral sequence (GenBank acc. no. AF074086.2; ref. [31]). Notably, transcripts from both loci employed a SA2 site located 7 nt downstream from the canonical SA2 site due to a mutation within that site. Transcripts assignable to the HERV-K111 provirus were identified in our study in all but four of the investigated normal tissue types including lack of detection of HERV-K111 transcript in PBL. The HERV-K111 provirus was previously reported to be specifically active during HIV infection [32].

Transcripts from the ‘Venter locus’ were identified in 11 of the investigated tissues. Since several of the employed tissue cDNA panels were pools from higher numbers of donors, it seems less likely that lack of transcripts in several tissues is due to a polymorphic presence/absence status of the HERV-K111 and the Venter locus in respective tissues. In any case, more detailed analyses will be required to characterize especially the status, genome location, and exact sequence of the Venter locus. We note in this context that numerous sequence variants of HERV-K111 were recently reported [34]. The Venter locus sequence displays 35-nt differences to HERV-K111 along 4.178 kb of comparable sequence and only 1-nt difference along the amplified cDNA sequence portion. While the sequence of the Venter locus is not identical to any of the reported HERV-K111 variants (data not shown), it is nevertheless conceivable that the Venter locus is, in fact, an undescribed variant of HERV-K111.

Additional analyses will also be required to identify the HML-2 source locus (or loci) producing an alternatively spliced, HML-2 type 1 locus-derived mRNA that utilizes a splice donor site located 252 nt downstream from the canonical *np9* mRNA’s SD2 and thus resulting in a longer mRNA. The isolated cDNA sequences display an open reading frame of 408 nt, starting at nt 41 of the cDNA sequences (nt 6805 relative to the HERV-K(HML-2.HOM) sequence, GenBank acc. no. AF074086), encoding a 135 aa long protein identical to HML-2 Env along the N-terminal 80 aa. Since our employed forward PCR primer overlapped with the start codon of the Env, Rec, and Np9 ORFs, we currently do not have information as to whether the source locus of the alternative splice variant has a start codon identical to the start codon of Env/Rec/Np9 proteins. That is, the ORF may extend further upstream from the start codon at nt 41 of the available cDNA sequence up to the Env/Rec/Np9 canonical start codon.

In this context, we also obtained cDNA sequence evidence for splicing of transcripts from a previously characterized HML-2 locus in human chromosome 10 very likely formed by reverse transcription and integration of a HML-2 transcript lacking *env* gene portions [21]. Testis- and colon-derived cDNA sequences included one sequence each lacking a 334-nt sequence compared to full-length cDNA sequences from that chromosome 10 locus. The missing sequence portion was compatible with that sequence being a spliced out intron. The resulting protein from that splice variant would encode a chimeric protein consisting of Rec and Np9 portions (Additional file 1: Figure S3).

## Conclusions

Our study demonstrates HERV-K(HML-2) *rec* and *np9* transcripts from various HML-2 loci in various normal human tissues. Among them are Rec or Np9 protein coding loci and it is thus conceivable that Rec and Np9 proteins might be present in normal human tissues. Rec/Np9/Env-like proteins potentially encoded by retrotransposed HERV-K(HML-2) loci identified recently and in this study may also be present in various normal tissues. Besides potential roles in disease development, it seems worthwhile hypothesizing that various HERV-K(HML-2)-encoded proteins exert biological functions also in normal human tissues. Better knowledge of specific HERV-K(HML-2) loci encoding *rec* and *np9* mRNAs in the various human tissue-composing cell types and knowledge of amounts of Rec and Np9 protein present in those cell types will likely contribute to a better understanding of those proteins’ functions under normal cellular conditions.

## Methods

### Multiple tissue cDNA panel and tissue total RNAs

We utilized the Human MTC™ Panel I and II (Clontech/Takara Bio, Otsu, Japan). The two panels included normalized, first-strand cDNA preparations from RNA from, in total, 15 different normal human tissues and peripheral blood leukocytes. cDNAs for each tissue consisted of pools from 3 to 98 Caucasian individuals and 550 male/female Caucasians in the case of peripheral blood leukocytes. Lung and liver derived cDNAs were from one male Caucasian each.

We also utilized commercially available total RNA from normal human heart, brain, and colon tissues (Clontech/Takara Bio; catalogue numbers 636532; 636530; 636553).

### Amplification of *np9* and *rec* PCR products, cloning of PCR products, plasmid preparation, sequencing

We amplified *rec* and *np9* mRNA representing PCR products from tissue cDNA panels employing forward primers rec-np9-for-1: 5′-ATG AAC CCA TCA GAG ATG CAA-3′; rec-np9-for-2: 5′-ATG AAT CCA TCA GAG ATG CAA-3′; rec-np9-for-3: 5′-GCG AAC CCT TCA GAG ATG CAA-3′; rec-np9-for-4: 5′-ATG AAC CCA TCG GAG ATG AAA-3′; that were combined in a ratio of 85/5/5/5, and reverse primers rec-np9-rev-1: 5′-AGC ATC TGT TTA ACA AAG CA-3′; rec-np9-rev-2: 5′-AGC ATG TTT AAC AAA GCA-3′ 5% combined in a ratio of 95/5. The various primer variants considered sequence differences of HERV-K(HML-2) loci within primer binding regions. PCR products were amplified using standard conditions with AmpliTaq Gold (Applied Biosystems/Life Technologies, Carlsbad, CA, USA) DNA polymerase and the following PCR program: 12 min 95°C; 35 cycles: 50 s 95°C, 50 s 58°C, 30 s 72°C, and final elongation 10 min 72°C. PCR products were separated by agarose gel electrophoresis; *np9* and *rec* representing PCR products were purified from gels using NucleoSpin Gel and PCR Clean-Up Kit (Macherey-Nagel GmbH & Co. KG, Düren, Germany). Products were cloned into pCR II-TOPO (Invitrogen/Life Technologies) and transformed into *Escherichia coli* DH-5α cells. Plasmid DNA from randomly selected bacterial colonies was purified and subjected to Sanger sequencing (see below).

We amplified *rec* and *np9* mRNA representing cDNA from total RNA from heart, brain, and colon tissues by RT-PCR following a previously established procedure [21]. PCR products were cloned into pGEM T-Easy (Promega GmbH, Mannheim, Germany). Plasmid DNA from randomly selected bacterial colonies was prepared as described before [32].

cDNA inserts were sequenced using vector-specific T7 primer and an Applied Biosystems 3730 DNA-Analyzer (Seq-IT GmbH, Kaiserslautern, Germany). Sequence qualities were verified by eye, and poor quality sequence reads were excluded from further analysis.

### Chromosomal assignment of cDNA sequences

We assigned *rec* and *np9* representing cDNA sequences to specific HML-2 loci by sequence comparisons essentially as described before [21,31]. Sequences that could be unambiguously assigned to a HML-2 locus in the human reference genome sequence with less than three mismatches were considered for analysis. Sequences not matching a locus in the human reference genome sequence are described in the main text. For assignment of *np9* cDNA sequences, we omitted the rather short (23 nt when excluding primer binding region) and thus little informative exon 2 of *np9* mRNA (SA1 - SD2) because the remaining sequence portions provided a sufficient number of sequence differences between (type 1) loci (see Additional file 1: Figure S1).

### Analysis of HML-2 loci for *np9* mRNA coding capacity

Similar to a recent analysis of HML-2 *rec* coding capacity [27], we examined in a multiple alignment of genomic HML-2 sequences, plus polymorphic HML-2 proviruses not present in the human reference genome sequence, features required for *np9* mRNA splicing and Np9 protein coding capacity, specifically presence of 5′ and 3′LTRs, splice donor and acceptor sites, and open reading frames as previously described [7].

## Availability of supporting data

LN624403 and LN624404 are accession numbers of cDNA sequences assignable to two HML-2 loci in human chromosomes 2q32.1 and 5q15 reported in this study and previously [21]. Accession numbers LN680257 to LN680271 are 245 bp longer, *np9*-like cDNA sequences. Sanger sequence reads generated in the course of this study have been deposited at the European Nucleotide Archive (study accession number PRJEB8273).

## Additional file

Additional file 1:
**Additional analysis of HERV-K(HML-2) sequences.** **Figure S1.** Sequence differences between *rec* and *np9* cDNA sequences. **Figure S2.** Structure of ‘Venter locus’. **Figure S3.** A spliced transcript encoded by a HERV-K(HML-2) locus in human chromosome 10.
