# Supplementary material for: HERV-K(HML-2) rec and np9 transcripts not restricted to disease but present in many normal human tissues
Source: Mob DNA. 2015 Mar 5;6:4. doi: 10.1186/s13100-015-0035-7 (PMC4351823; doi:10.1186/s13100-015-0035-7)
Supplement: Additional file 1: — Additional analysis of HERV-K(HML-2) sequences. Figure S1. Sequence differences between rec and np9 cDNA sequences. Figure S2. Structure of ‘Venter locus’. Figure S3. A spliced transcript encoded by a HERV-K(HML-2) locus in human chromosome 10. [file 13100_2015_35_MOESM1_ESM.pdf]

HERV-K(HML-2) *rec* and *np9* transcripts not restricted to disease but present in many normal human tissues

Katja Schmitt, Kristina Heyne, Klaus Roemer, Eckart Meese, Jens Mayer

Additional information

Additional Figure S1 - Locus-specific sequence differences for *rec* and *np9* mRNA sequences examined in this study.

Additional Figure S2 - HERV-K(HML-2) proviral portions in the "Venter locus".

Additional Figure S3 - A spliced transcript encoded by a HERV-K(HML-2) locus in human chromosome 10.

Rec

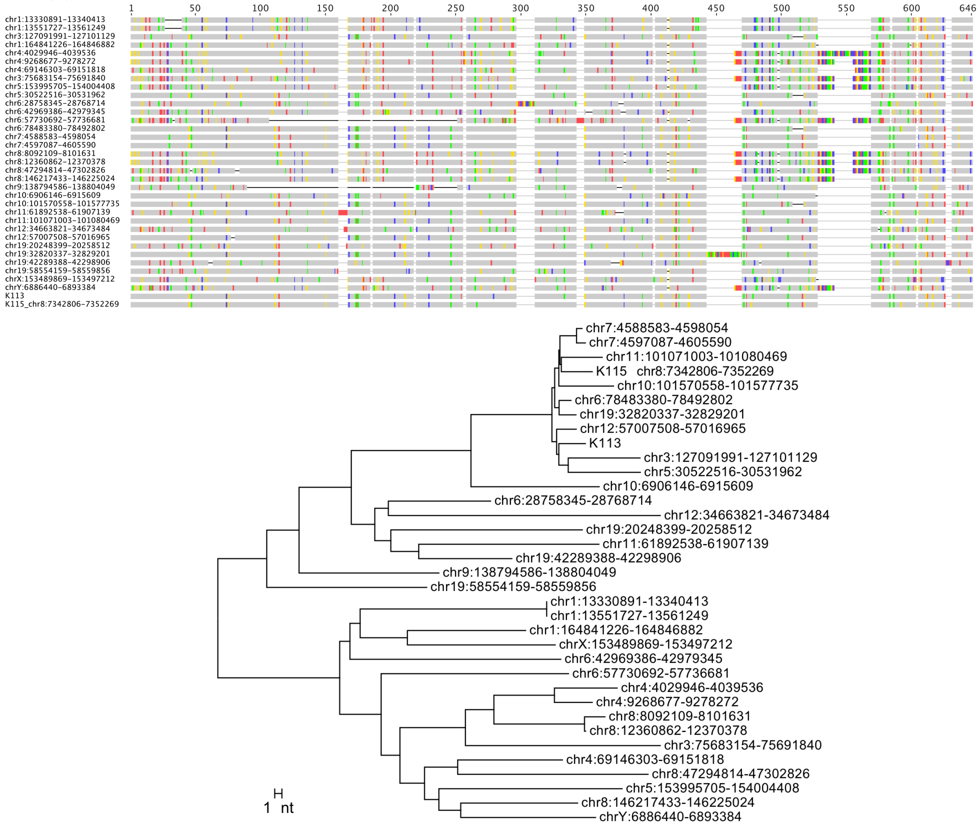

np9

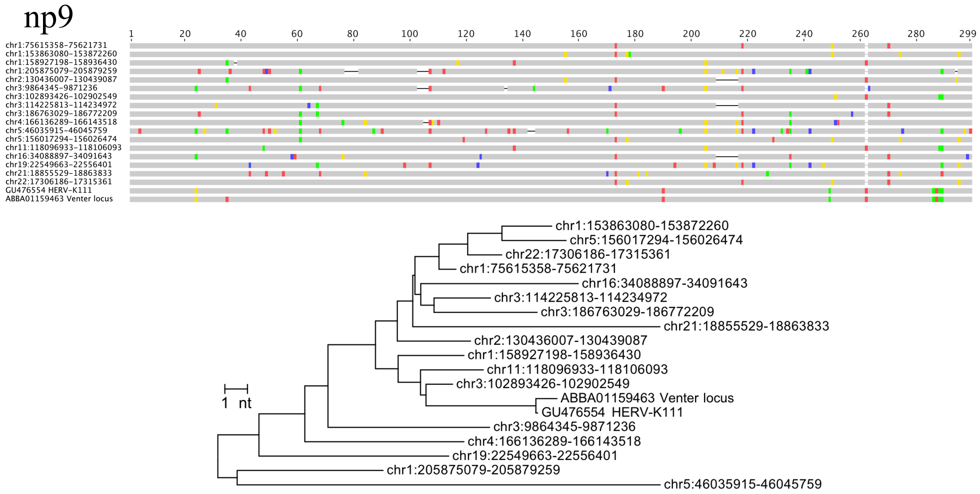

Additional Figure S1 - Locus-specific sequence differences for *rec* and *np9* mRNA sequences examined in this study.

HERV-K(HML-2) type 1 and type 2 loci were compiled from the human hg18 reference genome sequence and Genbank (see main paper text). *np9* and *rec* mRNA exon 2 and 3 portions, as amplified in our experimental design, were merged for each HML-2 locus and resulting sequences were multiply aligned. Nucleotide positions differing from the consensus sequence of included sequences are highlighted each. Primer binding regions were excluded from the alignments. Below each alignment are neighbour-joining trees depicting the absolute number of nt differences (disregarding indels) between HML-2 locus sequences amplified by RT-PCR documenting unambiguous assignment of cDNA sequences to HML-2 loci in almost all cases.

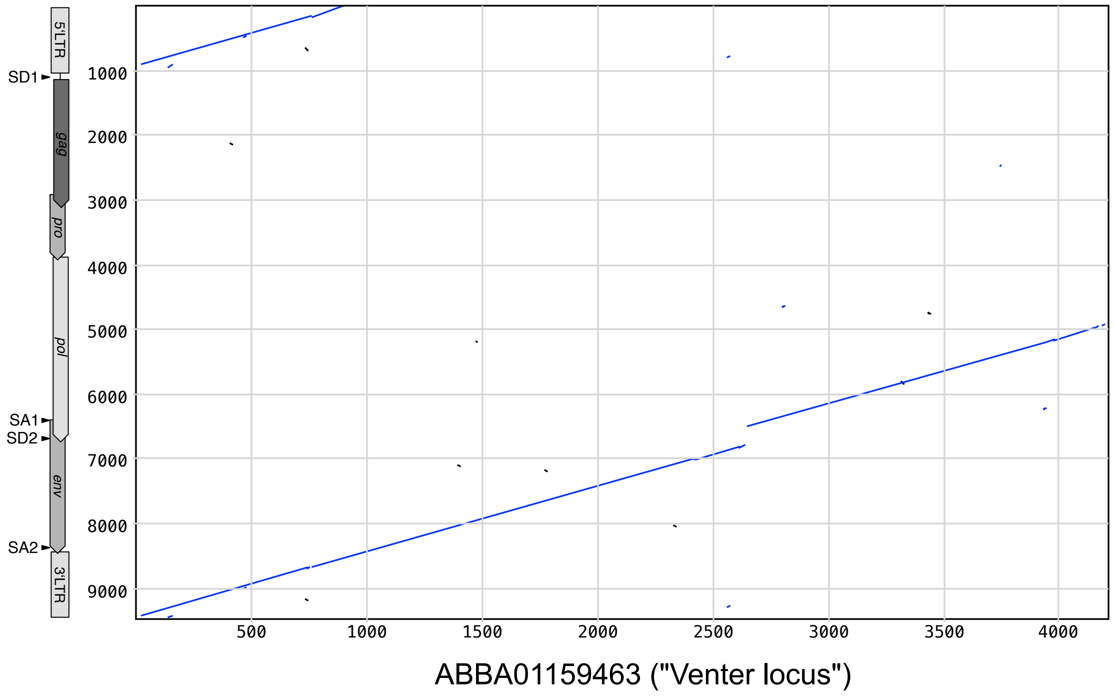

Additional Figure S2 - HERV-K(HML-2) proviral portions in the "Venter locus".
The Venter locus sequence entry [Genbank acc. no. ABBA01159463; ref. 1] was compared to the HERV-K(HML-2.HOM) provirus sequence as previously reported [Genbank acc. no. AF074086; ref. 2] using Pustell Matrix Comparisons as implemented in MacVector v13.5 with parameters: window size=20; min. % score=60; hash value=8; jump=1; strand=both.

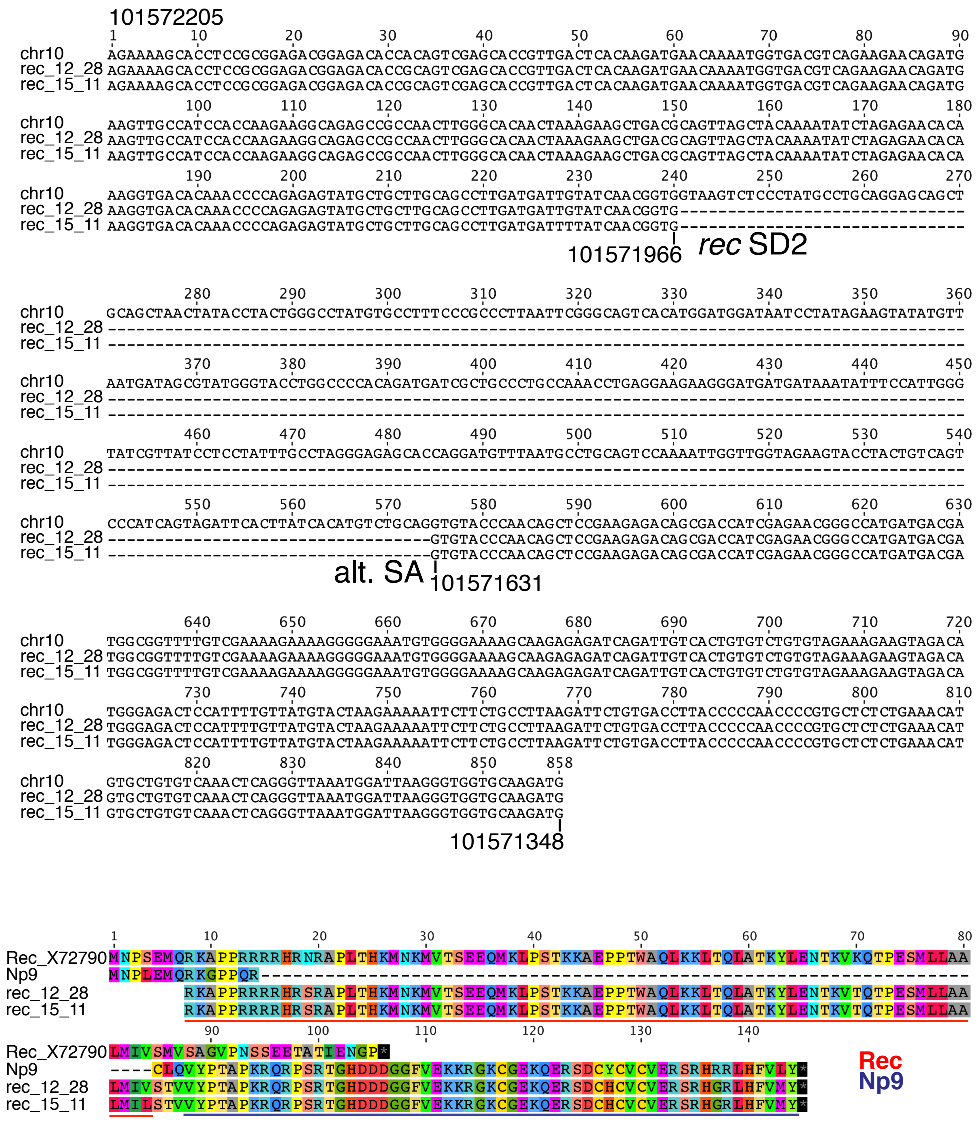

Additional Figure S3 - A spliced transcript encoded by a HERV-K(HML-2) locus in human chromosome 10.

Multiple alignment of a HERV-K(HML-2) locus located in human chromosome 10 (hg18; chr10:101570559-101577735), recently described as a partially spliced mRNA lacking an *env* gene region re-inserted as DNA copy into the genome [3], and two cDNA sequences (excluding primer regions) derived from testis (rec_12_28) and colon (rec_15_11) generated in the course of this project. Compared to the chromosome 10 locus, the two cDNA sequences lack a sequence region that is compatible with splicing of an intron, i.e. GT-AG borders, from a chromosome 10 locus precursor transcript. The splice donor site is identical with the one used for splicing of *rec* mRNA (*rec* SD2). An alternative splice acceptor site (alt. SA) has been used located 3 nt downstream of the canonical *rec*/*np9* SA2, the latter of which is missing in the chromosome 10 locus due to the splicing event before re-insertion [3]. Additional numbers give chromosome positions in human chromosome 10 with respect to the hg18 human reference genome sequence version. Note that the chromosome 10 locus sequence portions are given in reverse-completement orientation.

The multiple alignment at the bottom depicts protein translations of spliced cDNA sequences from the chromosome 10 locus compared to previously published Rec and Np9 protein sequences [4, 5]. Note that the presumable protein encoded by the alternatively spliced transcript resembles a chimera of Rec and Np9 proteins as indicated by underlined alignment portions. The N-terminal 7 amino acids are excluded from the presumable protein sequences as they overlap with the primer binding region for which the exact nucleotide sequence is currently not known.

References

1. Levy S, Sutton G, Ng PC, Feuk L, Halpern AL, Walenz BP, Axelrod N, Huang J, Kirkness EF, Denisov G *et al*: **The diploid genome sequence of an individual human**. *PLoS biology* 2007, **5**(10):e254.
2. Mayer J, Sauter M, Racz A, Scherer D, Mueller-Lantzsch N, Meese E: **An almost-intact human endogenous retrovirus K on human chromosome 7**. *Nat Genet* 1999, **21**(3):257-258.
3. Schmitt K, Reichrath J, Roesch A, Meese E, Mayer J: **Transcriptional profiling of human endogenous retrovirus group HERV-K(HML-2) loci in melanoma**. *Genome biology and evolution* 2013, **5**(2):307-328.
4. Armbruester V, Sauter M, Krautkraemer E, Meese E, Kleiman A, Best B, Roemer K, Mueller-Lantzsch N: **A novel gene from the human endogenous retrovirus K expressed in transformed cells**. *Clin Cancer Res* 2002, **8**(6):1800-1807.
5. Löwer R, Tönjes RR, Korbmacher C, Kurth R, Löwer J: **Identification of a Rev-related protein by analysis of spliced transcripts of the human endogenous retroviruses HTDV/HERV-K**. *J Virol* 1995, **69**(1):141-149.
